# Drug Fallacies

**Published:** 1906-12-29

**Authors:** 


### Drug Fallacies.

It is not an easy thing to bring drugs, as agents in the treatment of disease, to the bar of an impartial and conclusive tribunal. In the ordinary processes of treatment the attendant circumstances forbid the institution of the conditions essential to a critical scientific experiment. The immediate claim and duty is to employ all the means likely to bring relief, and to check the tendency to death. Thus the attempt to identify the exact part played by each of the agents employed is practically forbidden. When, as distinct from bedside observation, laboratory experiments are instituted, they, in their turn, are open to the damaging retort that what may be true in the case of such an animal as a frog or a cat is by no means true of human beings. Hence a judicious empiricism remains the ruling factor in treatment, and we are far from saying that in the present stage of our knowledge it is other than the safe and wise course. Yet it cannot be denied that this practice produces some very unsatisfactory and bewildering results. When, for example, two of the most eminent physicians and careful clinical observers of the day are in direct conflict upon such a comparatively simple and elementary therapeutic problem as the value of opium in pneumonia, it must surely be allowed that we have not yet reached a decisive method of estimating the actions and uses of drugs in the treatment of disease.

The present position of drug treatment being, in general at all events, so unsatisfactory, help from any quarter may well be welcomed. Even the so-called "practical" man can be none the worse, and may indeed be much the better, for the study of some of the results of experiments made in pharmacological laboratories. We are far from saying that such results ought to be set in successful opposition against clinical observations. The latter, it is true, often lack the quality of scientific precision, but they emerge from the actual problem, that is the treatment of the patient, and thus with them rests the final verdict. In short, whilst the pharmacologist is not to be set up as the arbiter of treatment, he may offer hints to the practical physician, and possibly may sometimes explain the conflict which expresses itself in connection with some of the most widely used drugs.

It is in the spirit just defined that attention may be directed to Professor W. E. Dixon's article on "Drug Fallacies." In this are presented some of the reasons for the differences of opinion which are stated by different physicians in regard to the values of individual drugs. Among these reasons must be recognised imperfect observation, and such fallacies as confusion of the *post* with the *propter hoc*. Of these and the like we do well to be warned, though we can hardly hope for their complete suppression. But there are grounds for error which are susceptible to more effective control. One of these is variability in the quantitative composition of medicinal preparations. The Pharmacopœia has fixed many standards, and full justice ought to be done to the manufacturing pharmacists for their sustained effort to realise these standards. But it still remains true that in many cases different specimens of one and the same preparation differ widely in the proportion of active ingredient which they contain. According to Professor Dixon, a remedy of the importance of digitalis is one of the greatest offenders in this respect. He does not hesitate to say that probably many hundreds of patients die every year in consequence of the fact that so many of the pharmaceutical preparations of digitalis and of other cardiac tonics do not possess the virtues of the drugs which they are supposed to represent. He finds much the same to be true of ergot, the preparations of which, as ordinarily sold, he has proved to possess but little of the action of the drug from which they are made. A more serious indictment of the efficiency of many of our modern pharmaceutical methods could not be framed, and it is vital that the charge should be pressed until it leads to the necessary reforms. Thus under existing conditions, it is manifest, from Professor Dixon's experiments, that in many instances, and these not the least important or least frequently employed, the physician, in prescribing a given preparation, has no guarantee that it really possesses the medicinal properties suggested by its name.

The improvement demanded by these results is the introduction of quantitative standards for all active medicinal preparations. In many instances where such standards can be determined by chemical estimation they have already been adopted in the Pharmacopœia. But digitalis, ergot, and numerous other drugs, cannot be valued by any known chemical tests. For them there remains only the application of physiological tests, and it is to the enforcement of these that Professor Dixon's paper points. Until such tests are adopted some of our best-known medicines may be empty of real value—a name and nothing more. It lies with the profession to see that the reform demanded is duly adopted.

